# Host‐mediated shift in the cold tolerance of an invasive insect

**DOI:** 10.1002/ece3.2564

**Published:** 2016-10-20

**Authors:** Amy C. Morey, Robert C. Venette, Erica C. Nystrom Santacruz, Laurel A. Mosca, W. D. Hutchison

**Affiliations:** ^1^ Department of EntomologyUniversity of MinnesotaSt. PaulMNUSA; ^2^ USDA, Forest ServiceNorth Central Research StationSt. PaulMNUSA

**Keywords:** *Epiphyas postvittana*, fitness, partial freeze tolerance, polyphagy, risk assessment, supercooling point

## Abstract

While many insects cannot survive the formation of ice within their bodies, a few species can. On the evolutionary continuum from freeze‐intolerant (i.e., freeze‐avoidant) to freeze‐tolerant insects, intermediates likely exist that can withstand some ice formation, but not enough to be considered fully freeze tolerant. Theory suggests that freeze tolerance should be favored over freeze avoidance among individuals that have low relative fitness before exposure to cold. For phytophagous insects, numerous studies have shown that host (or nutrition) can affect fitness and cold‐tolerance strategy, respectively, but no research has investigated whether changes in fitness caused by different hosts of polyphagous species could lead to systematic changes in cold‐tolerance strategy. We tested this relationship with the invasive, polyphagous moth, *Epiphyas postvittana* (Walker). Host affected components of fitness, such as larval survivorship rates, pupal mass, and immature developmental times. Host species also caused a dramatic change in survival of late‐instar larvae after the onset of freezing—from less than 8% to nearly 80%. The degree of survival after the onset of freezing was inversely correlated with components of fitness in the absence of cold exposure. Our research is the first empirical evidence of an evolutionary mechanism that may drive changes in cold‐tolerance strategies. Additionally, characterizing the effects of host plants on insect cold tolerance will enhance forecasts of invasive species dynamics, especially under climate change.

## 1 Introduction

In temperate environments, the inclement conditions of winter can be a strong selective force on insect life histories. In the face of subzero temperatures and the potentially lethal effects of ice formation, insects generally either avoid freezing (a strategy known as *freeze avoidance*, resulting from freeze intolerance) or tolerate it under certain conditions (*freeze tolerance*) (Lee, 2010). Passionate discussion has surrounded the importance of gradients within and between these strategies, and how responses to cold could be further delineated (e.g., Bale, 1996; Chown, Sørensen, & Sinclair, 2008; Nedvěd, 2000; Sinclair, 1999). Broadly, though, evolutionary and geographic relationships distinguish groups that withstand freezing and those that do not (Addo‐Bediako, Chown, & Gaston, 2000; Sinclair, Addo‐Bediako, & Chown, 2003), with most evidence supporting freeze tolerance as a derived state from freeze avoidance (Costanzo & Lee, 2013; Vernon & Vannier, 2002; but see also Chown & Sinclair, 2010).

Evolutionary intermediates are likely to exist between freeze avoidance and tolerance (e.g., Overgaard, Sorensen, & Loeschcke, 2010). These intermediates are likely to have complex relationships between temperature, time, freezing, and death (Bale, 1993). Some may survive exposure to the temperature of crystallization (i.e., the supercooling point, the temperature at which an insect's body fluids begin to freeze), but not survive when equilibrium ice formation is achieved or for prolonged periods (i.e., >24 hr) in a semifrozen state (Block, Erzinclioglu, & Worland, 1988; Hawes & Wharton, 2010; Sinclair, 1999). Sinclair (1999) classifies these insects as “partially freeze tolerant,” but acknowledges that it is uncertain whether survival of partial freezing “represents the norm among freeze‐avoiding insects that die at the supercooling point” or is a unique freeze tolerance strategy. Although the immediate ecological relevance of partial freeze tolerance is uncertain, it may be more germane to evolutionary questions of insect cold tolerance; identifying and characterizing such evolutionary intermediates may provide valuable insights into how species adapt and evolve to withstand low temperatures (Sinclair, 1999; Voituron, Mouquet, de Mazancourt, & Clobert, 2002).

Freeze tolerance has evolved independently multiple times among insects, and many factors may drive the change, such as climatic predictability (Sinclair et al., 2003), behavioral and physiological mechanisms that affect ice nucleation (Duman, Wu, & Xu, 1991; Lee & Costanzo, 1998; Strachan, Tarnowski‐Garner, Marshall, & Sinclair, 2011; Zachariassen, 1985), energy conservation from altered metabolic rates (Irwin & Lee, 2002), and cross‐tolerance with other environmental stressors (e.g., Holmstrup, Bayley, Pedersen, & Zachariassen, 2010; Sinclair, Ferguson, Salehipour‐Shirazi, & Macmillan, 2013). Of note, Voituron et al. (2002) developed a formalized model to explain conditions under which a particular cold‐tolerance strategy should be optimal, based on an energetic definition of fitness. Freeze avoidance is energetically costly, so the authors concluded that freeze‐tolerant individuals would be favored when the fitness level before exposure to cold is low, whereas freeze‐avoidant individuals would be favored when fitness is initially high. No empirical evidence currently exists to support this hypothesis, although Convey (2010) points to life history trade‐offs as a consequence of the greater energetic costs associated with the production of cryoprotectants in freeze‐avoidant arthropods. Evaluating cold stress in species with intermediate cold‐tolerance phenotypes, such as those showing partial tolerance to freezing, may be particularly insightful to show adaptations to freezing stress (Voituron et al., 2002).

For phytophagous insects, especially those that are polyphagous, host plant (hereafter, “host”) may significantly affect fitness (Awmack & Leather, 2002; Danthanarayana, 1975a; Röder, Rahier, & Naisbit, 2008) and cold tolerance. Host has changed supercooling points and cryoprotectant levels in a number of insect species (Gash & Bale, 1985; Liu et al., 2009; Verdú, Casas, Lobo, & Numa, 2010; but see also Kleynhans, Conlong, & Terblanche, 2014; Rochefort, Berthiaume, Hébert, Charest, & Bauce, 2011). Trudeau, Mauffette, Rochefort, Han, and Bauce (2010) noted that seemingly poor‐quality hosts yielded the highest overwintering success in *Malacosoma disstria* (Hübner). In contrast, Liu et al. (2009) found poor developmental hosts produced low overwintering survival in diapausing *Helicoverpa armigera* (Hübner). However, none of these studies were designed to address host influences on cold‐tolerance strategy.

We previously documented variation in the ability of the light brown apple moth, *Epiphyas postvittana* (Walker), to survive the onset of freezing (Morey, Venette, & Hutchison, 2013). *Epiphyas postvittana* is a recent invader to the continental United States (Brown, Epstein, Gilligan, Passoa, & Powell, 2010) with the capacity to feed on over 360 plant genera (Suckling & Brockerhoff, 2010). For this species, host is known to affect immature survival, developmental time, and pupal weight (Danthanarayana, 1975b; Tomkins, Penman, & Chapman, 1989), which are common proxies of ecological fitness (Via, 1990). This species predominantly overwinters as late‐instar larvae, which do not enter diapause and are generally considered freeze avoidant (Buergi, Roltsch, & Mills, 2011; Bürgi & Mills, 2010). However, we observed that a small proportion (~20%) of late instars from a laboratory population could survive the initial formation of ice within their bodies from exposure to their supercooling points. Moreover, many of the survivors continued development and eclosed as reproductively successful adults (Morey et al., 2013). Given the extensive polyphagy of the species and the putative ability of some individuals to withstand brief exposure to internal freezing, we sought to examine the extent to which host systematically affected the cold‐tolerance strategy of *E. postvittana*.

Our study revealed that hosts can dramatically affect the cold response of this species. Across five hosts, we found a nearly 10‐fold range in the survival of *E. postvittana* larvae after exposure to their supercooling points. These hosts also affected survivorship rates and developmental times in the absence of cold. Most importantly, we found that as components of fitness decreased, the extent of survival to partial freezing increased, thus supporting the hypothesis of Voituron et al. (2002) that low initial fitness may favor a shift toward freeze tolerance.

## 2 Materials and Methods

### 2.1 Insect colony and plant materials

*Epiphyas postvittana* eggs were obtained in 2012 and 2013 from a laboratory colony maintained by the United States Department of Agriculture, Animal and Plant Health Inspection Service (USDA‐APHIS) in Albany, CA, founded from wild California moths in 2007. All subsequent handling and experimentation was conducted in a Biosafety Level 2 Containment Facility in St. Paul, MN (APHIS permit P526P‐14‐03759). Eggs were surface‐sterilized in a 1% bleach solution and held at 23 ± 2°C, 60%–65% RH, 14:10 (L:D) inside a growth chamber (Percival Scientific, Perry, IA) until hatch.

Host treatments were selected from a list of documented North America hosts for *E. postvittana* (Venette, Davis, Dacosta, Heisler, & Larson, 2003), with priority given to those species and varieties that occur in temperate (i.e., Midwestern United States) climates. The following phylogenetically diverse hosts were used during the experiment: *Vitis vinifera* L. (var. “Frontenac”), *Malus domestica* Borkh. (var. “Zestar!”), *Pinus banksiana* Lamb., and *Populus deltoides* ssp. *monilifera* (Aiton) Eckenwalder (var. “Siouxland”). *Vitis vinifera* were potted as cuttings from the University of Minnesota Horticultural Research Center (Excelsior, MN). *Pinus banksiana* used in 2012 (blocks 1 and 2) were potted as seedlings from the Minnesota Department of Natural Resources Badoura State Forest Nursery (Akeley, MN). *Pinus banksiana* in 2013 (block 3) were from mature trees planted on the University of Minnesota campus (St. Paul, MN). *Malus domestica* and *Po. deltoides* were planted as 2‐ to 3‐year‐old nursery stock trees in the University of Minnesota campus (St. Paul, MN). All potted plants were housed in a greenhouse during winter months (September–May) and moved outdoors during summer months. No pesticides were applied. An artificial diet was used as control; the diet used *Phaseolus vulgaris* L (cv. Great Northern) and followed a modified formulation developed by Follett and Lower (2000) for a related species. Our modifications included the following: doubling the recipe ingredients, adding a total of 2034 ml of water, and autoclaving all heat stable products together for 45 min at 121°C and 103.4 kPa.

Within 24 hr of hatching, *E. postvittana* neonates were placed individually onto a randomly selected host using surface‐sterilized (with 70% ethanol in water) camel hair paintbrushes. Groups of three larvae were placed on a single excised leaf (or sprig, in the case of *Pinus*), which was contained in a Petri dish sealed with parafilm to prevent larval escape. Leaf petioles were inserted into 1 cm^3^ of wet floral foam, which was rewetted with deionized water every 2–3 days. Leaves were replaced on average every 3 days when discoloration was observed, or larvae consumed ~1/2 of the tissue area. Larvae were similarly applied to ~2 cm^3^ of artificial diet in sealed plastic cups (29.5‐ml P100 soufflés; Solo Cup Co., Lake Forest, IL). 20–26 dishes (or cups) were set up for each host treatment per block, except for block three of *Pi. banksiana* which required a total of 45 dishes due to poor initial viability of neonates (Table S1). Larvae remained on their respective host treatments until the time of cold exposure.

### 2.2 Cold exposure

Instar was confirmed through head capsule measurement (Danthanarayana, 1975a), and late instars (4th–6th) from each host were randomly assigned to one of two temperature treatments, cooled to the point of producing an exotherm (i.e., the supercooling point) or not cooled (i.e., temperature control). *Epiphyas postvittana* is known to have variable instar numbers (Danthanarayana, 1975a; Dumbleton, 1932). Therefore, we focused on “late instars,” any of which could be the terminal instar before pupation and could overwinter. The number of larvae in each temperature treatment depended on the mortality of early instars from a given host. A total of 8–32 larvae were tested per temperature treatment (*n* = 2) per host (*n* = 5) per block (*n* = 1–3). For the cold‐exposed groups, specifically, the total number of late instars tested in each host, across all blocks, was as follows: artificial diet, *n* = 64; *M. domestica, n* = 26; *Pi. banksiana*,* n* = 48, *Po. deltoides, n* = 38; *V. vinifera, n* = 64.

All larvae were transferred to individual gelatin capsules (size 4; 14.3 mm length, 5.1 mm diameter). For the supercooling treatment, individuals were cooled at ~1°C/min to their supercooling point within calibrated polystyrene cubes inside a −80°C freezer, as per Carrillo, Kaliyan, Cannon, Morey, and Wilcke (2004). Insect body temperatures were recorded once per second using a coiled, copper‐constantan thermocouple design (Hanson & Venette, 2013), connected to a computer through a multichannel data logger (USB‐TC; Measurement Computing, Norton, MA). Each larva was immediately removed from the freezer and cube once they reached the peak of the exotherm, which could be observed in real time as a plateau (typically lasting 15–25 s) following an abrupt spike in temperature. Larvae were then given fresh material of the host on which they were reared and returned to 23 ± 2°C (60%–65% RH, 14:10 [L:D]) in a growth chamber to continue development. Temperature control individuals were left inside gel capsules at room temperature (~25°C) for approximately 1 hr while cold‐treated individuals were being chilled. Temperature controls were removed from the capsules and given fresh diet concomitantly with the supercooled larvae. Survival was monitored daily for 5 weeks or until the individual eclosed as an adult (nothing emerged after 4 weeks).

### 2.3 Host suitability

To assess the developmental suitability of each host for *E. postvittana* without temperature stress, multiple developmental parameters, each a component of fitness, were measured for individuals that had not been exposed to cold: three stage‐specific survival proportions, pupal mass, and total developmental time. Proportion survival was assessed from neonate to late instar, from late instar to pupation, and from pupation to adult eclosion. Because temperature treatments were not assigned until larvae reached late instars, the proportion of survival to late instar included all individuals initially reared on a given host (i.e., irrespective of future cold treatment). Once individuals were divided among temperature treatments as late instars, only control larvae were used to assess the effect of host on survival during the two subsequent developmental periods, pupal mass, and total developmental time. Pupae were weighed 3–5 days after pupation. Total developmental time (egg hatch to adult eclosion) was only calculated for those that survived to adult eclosion.

### 2.4 Analysis

Analyses were conducted in SAS^®^ 9.4 (SAS Institute Inc., Cary, NC, USA). The experiment followed an incomplete block design, with three total blocks occurring in February 2012, March 2012, and August 2013. Due to changes in availability of plant material, the same host species were not included during every block (see Table S2). Sex was not considered in any analysis because the sexes of larvae could not be differentiated; equal sex ratios could not be assumed at the outset of the experiment.

The effects of host on all continuous response variables (i.e., supercooling point, pupal mass, total developmental time) were analyzed using mixed‐effects models (Proc GLIMMIX), with host treated as a fixed effect and block treated as a random intercept. For all variables, Levene's tests revealed unequal variances among some treatment groups, so treatment variances were grouped (GROUP = host) to account for heteroscedasticity (Littell, Milliken, Stroup, Wolfinger, & Schabenberger, 2006). Variances were estimated using restricted maximum likelihood (REML) due to the unbalanced design, and degrees of freedom were approximated by the Kenward–Roger method (Spilke, Piepho, & Hu, 2005). Treatment means were estimated with least‐squares means (Spilke et al., 2005) and differences were separated using Tukey–Kramer adjustments for multiple comparisons to maintain an overall α = .05.

Survival measures were also analyzed using a mixed‐effects model, but with a binomial distribution and logit link function (Proc GLIMMIX; events/trials syntax) to compare survival across hosts. Host was treated as a fixed effect, and block treated as a random effect. Degrees of freedom were estimated using the Kenward–Roger method, and Tukey–Kramer groupings were used to compare differences in the least‐squares means (Littell et al., 2006). Before comparing host effects on survival after partial freezing, an Abbott's correction (Rosenheim & Hoy, 1989) was applied to the response to account for control mortality in each host treatment.

The potential relationship between the temperature at which freezing occurred and the likelihood of surviving freezing was assessed using a mixed‐effects model as before. Here, host, state (dead or alive), and their interaction were used as explanatory variables of supercooling points. Block was treated as a random effect, and Tukey–Kramer groupings were used to compare the supercooling points of individuals that died to those that survived.

Multiple regression with backwards elimination was used to evaluate the relationship(s) between the cold stress response and host suitability measures. Cold stress response was the proportion of individuals that survived partial freezing (corrected for control mortality). Host suitability was reflected in the five fitness components (i.e., survival from hatch to late instar, survival from late instar to pupation, survival from pupation to adult eclosion, pupal mass, and total developmental time). Because we were interested in the broad impact of host suitability on survival after partial freezing, we did not distinguish host species in this analysis. Instead, we treated the response from each host/block combination as a distinct host measure. Before model construction, we tested for correlations among candidate predictors to ensure assumptions of regression were met. Mean pupal mass and mean total developmental duration were found to be negatively correlated (*P* = .016, F_1,9_ = 8.68). All other parameters were not significantly correlated with one another. So, two sets of candidate predictors were evaluated: The first initially included the three, stage‐specific survival proportions and total developmental time, and the second initially included the stage‐specific survival proportions and pupal mass. Regressions were run as mixed‐effect models (Proc GLIMMIX; events/trials syntax), with the developmental parameters treated as fixed effects, and block treated as a random effect. Degrees of freedom were estimated by the Kenward–Roger method. Backwards elimination was stopped once each model could not be further improved (i.e., all predictors were significant at *P* < .05).

## 3 Results

### 3.1 Supercooling and survival after partial freezing

**Figure 1:**
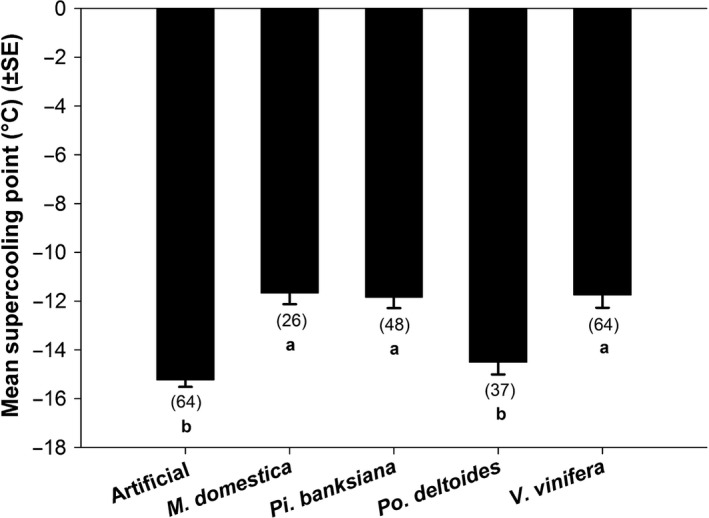
Mean supercooling points (±SEM) of late‐instar *Epiphyas postvittana* reared on different hosts. Least‐squares estimates are presented to account for an unbalanced mixed‐effects design. Bars with the same letter are not significantly different at α = .05. Numbers in parentheses indicate the total sample size across all blocks

Host affected the supercooling points of late instars (*P* = .0003, *F*_4,9.36_ = 16.75). Larvae fed artificial diet and *Po. deltoides* had significantly higher mean supercooling points (−15.23°C ± 0.29 and −14.51°C ± 0.50, respectively; mean ± SEM), than those fed *Pi. banksiana* (−11.84°C ± 0.45), *V. vinifera* (−11.75°C ± 0.53), and *M. domestica* (−11.67°C ± 0.46) (Figure 1).

**Figure 2:**
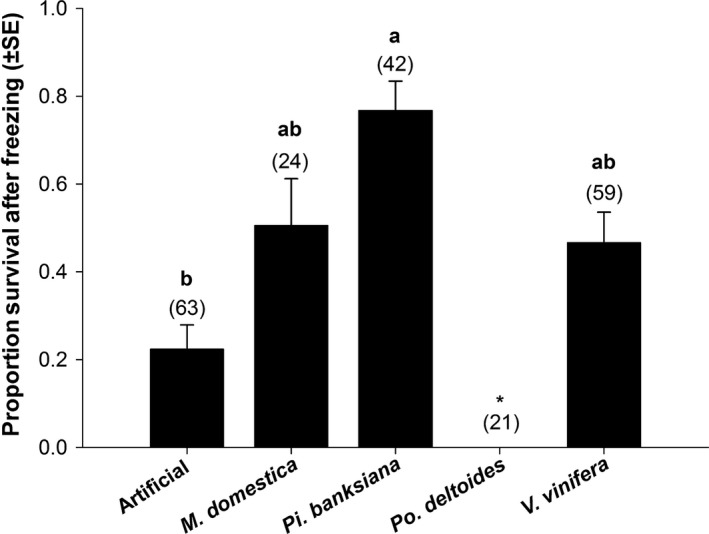
Mean proportion survival (±SEM) of late‐instar *Epiphyas postvittana* reared on different hosts following partial freezing. Survival was defined as successful adult eclosion. Bars with the same letter are not significantly different at α = .05. Numbers in parentheses indicate the total sample size across all blocks. *No larvae survived partial freezing when fed *Populus deltoides*

Survival to adult eclosion after partial freezing (i.e., exposure to the peak of the exotherm) was also affected by host (*P* < .015, *F*_3,6_ = 8.27). No late instars survived to adult eclosion after partial freezing when reared on *Po. deltoides,* so this host was not included in the subsequent means comparison. Larvae fed artificial diet survived in the next lowest proportion (0.22 ± 0.055). In contrast, survivorship was greatest for larvae reared on *Pi. banksiana* (0.77 ± 0.067). Those fed *M. domestica* and *V. vinifera* showed intermediate and statistically no different, partial freeze tolerance to the other hosts with survivors (0.51 ± 0.11 and 0.47 ± 0.069, respectively) (Figure 2).

**Figure 3:**
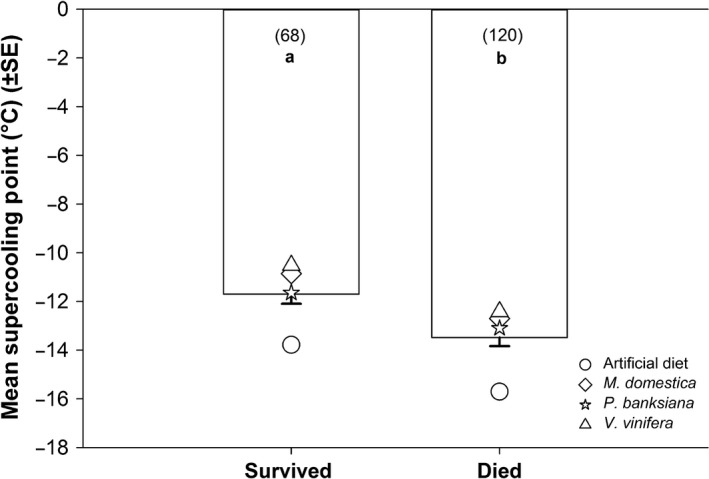
Relationship between survival and mortality after partial freezing and mean supercooling points in *Epiphyas postvittana* late instars reared on different hosts. Survival was defined by successful adult eclosion. Bars with the same letter are not significantly different at α = .05. Numbers in parentheses indicate the total sample size across all blocks. Symbols represent the mean supercooling point across all blocks for a given host

Across hosts, individuals that died after partial freezing had lower supercooling points than those that lived (*P* < .0001, *F*_1,178.8_ = 20.78) (Figure 3). As shown in the previous analysis, supercooling points were affected by host alone (*P* < .0001, *F*_3, 172.3_ = 18.18); the analysis output here differs slightly because *Po. deltoides* was not included in this model due to lack of survivors. Supercooling points were not affected by the interaction between host and state (dead or alive) (*P* = .96, *F*_3,179.2_ = 0.09).

### 3.2 Host suitability: stage‐specific survival, total immature developmental time, and pupal mass without freezing stress

Host affected the proportion of larvae that survived to be late instars (*P* < .0001, *F*_4,6_ = 60.55). Nearly all larvae survived to late instars when reared on artificial diet (0.98 ± 0.012), whereas those fed *Pi. banksiana* had the lowest survival (0.36 ± 0.13). Larvae fed *M. domestica, Po. deltoides,* and *V. vinifera* survived in statistically equivalent proportions (0.86 ± 0.076, 0.83 ± 0.089, and 0.88 ± 0.066, respectively) (Table 1). In contrast, host did not affect the survival of late instars to pupae (*P* = .08, *F*_4,6_ = 3.60), nor of pupae to adult eclosion (*P* = .65, *F*_4,4.7_ = 0.65) (Table 1).

**Table 1:** Summary of metrics used to define the suitability of five larval hosts of *Epiphyas postvittana*

Host	Proportion Survival^a^			Mean pupal mass (mg)	Mean total developmental time (d)^b^
	Hatch to late instar	Late instar to pupation	Pupation to adult eclosion		
Artificial diet	0.98 ± 0.001 (186)^a^	0.99 ± 0.0075 (96)^a^	0.95 ± 0.048 (95)^a^	37.8 ± 1.0 (94)^a^	36.5 ± 0.8 (90)^b^
*Malus domestica*	0.86 ± 0.076 (75)^b^	0.95 ± 0.060 (26)^a^	0.71 ± 0.33 (21)^a^	30.8 ± 6.1 (18)^ab^	39.1 ± 4.1 (15)^ab^
*Pinus banksiana*	0.36 ± 0.130 (254)^c^	0.98 ± 0.025 (33)^a^	0.97 ± 0.039 (32)^a^	31.9 ± 1.7 (32)^ab^	49.6 ± 2.5 (31)^a^
*Populus deltoides*	0.83 ± 0.090 (75)^b^	0.95 ± 0.065 (24)^a^	0.74 ± 0.32 (19)^a^	31.0 ± 2.3 (18)^ab^	34.6 ± 1.5 (14)^b^
*Vitis vinifera*	0.88 ± 0.066 (198)^b^	0.92 ± 0.094 (100)^a^	0.87 ± 0.11 (87)^a^	24.4 ± 2.2 (74)^b^	49.9 ± 0.2 (73)^a^

Data were collected from individuals that did not experience cold stress. Means (±SEM) are presented as least‐squares estimates to account for an unbalanced mixed‐effect design. Numbers in parentheses indicate total sample size across all blocks. Cells within a column with the same letter are not significantly different (*P* > .05).

a Sample size indicates the number of individuals going into a given developmental period.

b Time from egg hatch to adult eclosion; only measured for those that survived to adult eclosion.

Host affected pupal mass (*P* = .0009, *F*_4,16.1_ = 8.07). Artificial diet produced, on average, the pupae with the greatest mass (37.8 ± 1.0 mg) whereas *V. vinifera* produced pupae with the least mass (24.4 ± 2.2 mg). The pupal masses of insects reared on *Pinus banksiana, Po. deltoides,* and *M. domestica* were not significantly different from one another or the other two hosts (Table 1).

Host also affected the time from egg hatch to adult eclosion of *E. postvittana* (*P* < .0001, *F*_4,218_ = 49.68). Insects fed *V. vinifera* and *Pi. banksiana* took an average of nearly 50 days (49.9 ± 0.2 and 49.6 ± 2.5, respectively) to develop, whereas those fed artificial diet and *Po. deltoides* developed within an average of <37 days (36.5 ± 0.8 and 34.6 ± 1.5, respectively). *Malus domestica*‐fed larvae developed for an intermediate duration of 39.1 (±4.1) days, which was not statistically different from either extreme group (Table 1).

### 3.3 Relationship between host suitability and cold stress response

**Figure 4:**
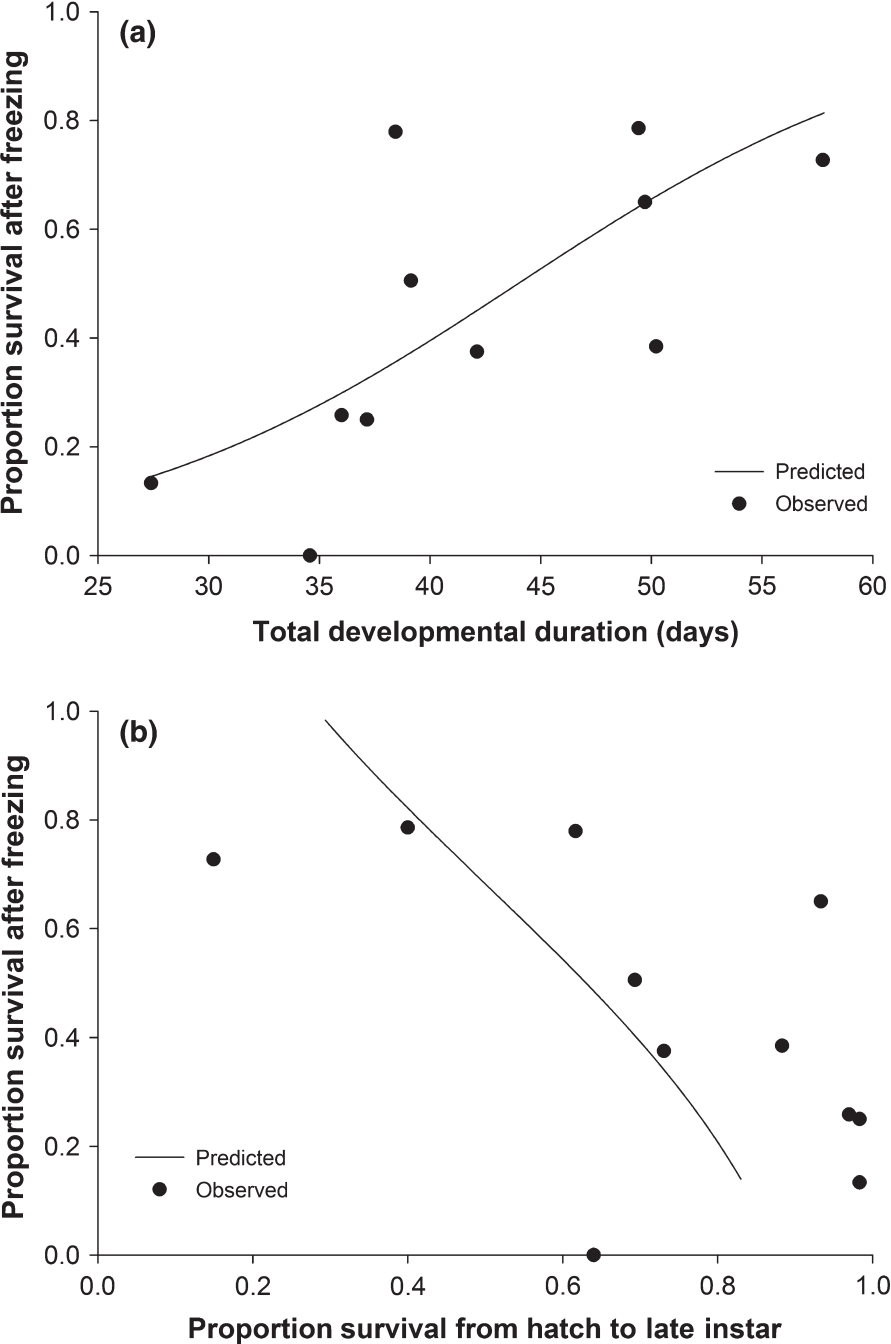
Effect of host quality (as represented by two components of fitness in individuals without cold exposure) on survival after partial freezing in late instars of *Epiphyas postvittana*. The predicted lines represent the results of logistic regressions relating the proportion of survival after partial freezing with (a) total immature developmental time (*F*_1,9_ = 21.73, *P* = .0012) and (b) the proportion of individuals that survived from hatch to late instar (*F*_1,9_ = 16.50, *P* = .0028) in non‐cold‐exposed populations. Points represent values of each block within each host treatment

Both regression models showed that the proportion of larvae that survived the onset of freezing was greater on less suitable hosts. Each model reduced to a single predictor from four candidate predictors and followed the form:

$$P\left(x\right)=\frac{1}{1+e^{-(b_{0}+b_{1}x)}}$$

where *P(x)* is the proportion of individuals with fitness level *x* (for the relevant fitness measure) that survived the onset of ice formation. In the first model, the proportion of individuals that survived the onset of ice formation was positively related to mean total developmental time (*P* = .012, *F*_1,9_ = 21.73) (Figure 4a). For this model, *b*_0_ = −4.70 (±1.01) and *b*_1_ = 0.11 (±0.02). In the second model, the proportion of individuals that survived the onset of ice formation was negatively related to the proportion of larvae that survived from hatch to late instar (*P* = .0028, *F*_1,9_ = 16.50) (Figure 4b). Here, *b*_0_ = 1.99 (±0.67) and *b*_1_ = −2.92 (±0.72). Host suitability, and by definition ecological fitness, decreased as developmental time increased, or larval survivorship decreased.

## 4 Discussion

We documented a nearly 10‐fold change in the survival of *E. postvittana* larvae following brief exposure to freezing due to the host on which larvae develop (Figure 2). These large, host‐mediated effects followed predictions that link the fitness expressed in temperature conditions without cold stress to the likelihood of exhibiting one cold‐tolerance strategy over another (Voituron et al., 2002) (Figure 4). The ability to survive the onset of freezing greatly increased among hosts that might otherwise be considered poor quality, specifically those hosts that induced the longest developmental time and allowed fewer larvae to reach late instars. The potentially counterintuitive relationship we observed between components of fitness and cold tolerance could be driven by the metabolic costs associated with different cold‐tolerance strategies (Voituron et al., 2002). The production of cryoprotectants to stave off freezing may be energetically costly (e.g., Convey, 2010), so if fitness is already low, theory suggests the ability to tolerate ice formation should be favored over freeze avoidance. Although we do not yet know the exact mechanism through which host is operating in our study, this pattern between fitness and cold response is what we observed in our data.

Our experimental results also highlight the significant plasticity that a species may exhibit in its cold‐tolerance strategy. Differences in cold‐tolerance strategy among species, among life stages of the same species, and among seasons for the same life stage are well documented. The Voituron et al. (2002) model suggests the possibility that, for a species at any moment, more than one cold‐tolerance strategy may be operating. Hawes and Bale (2007) review evidence for such mixed strategies and comment on this bet‐hedging strategy as a complement to plasticity in insect cold hardiness. In our case, although none of the late‐instar larvae of *E. postvittana* could be considered freeze tolerant, substantial variation existed in the proportion of individuals that died after the onset of freezing (little acute, prefreeze mortality occurs in late instars for this species; R.C. Venette, unpublished). This result suggests that the species may display a combination of freeze avoidance and some other response, perhaps “partial freeze tolerance.” Variation in cold‐tolerance response within a population is a precondition for the evolution of cold‐tolerance strategies.

Supercooling points also changed with host (Figure 1) in a direction that would be expected if a shift toward freeze tolerance were to occur; generally, freeze‐tolerant species supercool at warmer temperatures than freeze‐avoidant species (Lee, 2010; Sømme, 1982). For example, the larvae that showed the lowest survival after partial freezing, that is, those reared on artificial diet or *Po. deltoides* (which showed no survival), had the lowest mean supercooling points. Moreover, larvae fed *Pi. banksiana* had higher supercooling points and a significant relative increase in survival after partial freezing. This relationship is further supported by looking across hosts; larvae that survived partial freezing had higher supercooling points than those that died (Figure 3). Host species changed where freezing initiation began, but they did not change the proportional relationship between the supercooling points of survivors and nonsurvivors.

The changes in cold‐tolerance response of late‐instar *E. postvittana* that were caused by different hosts, although consistent with the model proposed by Voituron et al. (2002), do not preclude involvement of additional or alternative mechanisms. The nutritional content of the host plant may cause a direct impact on the cold‐tolerance response, in addition to indirect effects mediated through energy metabolism. Many of the biochemical mechanisms that protect individual insects from the damaging effects of cold depend upon the synthesis and accumulation of low molecular weight polyols (Storey & Storey, 1991), sugars (Bale, 2002), fatty acids (Koštál, 2010), or proteins (Duman, Xu, Neven, Thursman, & Wu, 1991). These products, or their precursors, are often accumulated as a result of the type and quality of the food being consumed. For phytophagous insects, especially those with a wide host range, cold tolerance could therefore differ substantially among individuals or populations, depending on the nutritional content of the host consumed. The few studies that have addressed dietary effects on cold‐tolerance mechanisms predominantly focus on artificially augmenting specific components of insect nutrition, such as amino acids (Koštál, Šimek, Zahradníčková, Cimlová, & Štětina, 2012), proteins (Andersen, Kristensen, Loeschcke, Toft, & Mayntz, 2010), cholesterol (Shreve, Yi, & Lee, 2007), sugar (Colinet, Larvor, Bical, & Renault, 2012), and nitrogen (Lavy & Verhoef, 1997), or on host effects of indirect measures of cold tolerance, such as diapause (Hunter & McNeil, 1997) and through tritrophic interactions (Li, Zhang, Zhang, Chen, & Denlinger, 2014).

Even fewer studies directly assess cold tolerance under different host plant environments. Our finding that less developmentally suitable hosts may favor increased resistance (or recovery) from ice formation provides a useful foundation to justify further exploration: for example, characterizing the physiological mechanisms of host effects, such as the nature of ice nucleation, the extent of ice formation, water content, and cryoprotectant systems across hosts that may affect cold‐tolerance response. Additionally, conducting assays under acclimatizing conditions (e.g., slower cooling rates, changes in photoperiod and developmental temperature regime) and with populations more recently out‐crossed with wild individuals would give insight as to the ecological implications of our results. Lastly, larval feeding cessation prior to overwintering could affect the supercooling point by altering potential ice nucleation sites in the gut (Sømme, 1982). While we did not determine feeding status of larvae at the time of cold exposure, large sample sizes and randomized specimen selection compensated for such potential effects. Moreover, *E. postvittana* larvae may continue to feed and develop during the winter months (Buergi et al., 2011; Geier & Briese, 1980) so variation in the presence of gut nucleators is likely to be present in field individuals.

Our study offers exciting implications for both theoretical and practical areas of ecology. By demonstrating that an increase in partial freeze tolerance is favored when initial components of fitness are low, we give preliminary experimental support to the larger hypothesis that more extensive freeze tolerance may also be favored in such conditions. We also provide further evidence of putative partial freeze tolerance in an insect, characterizing its tolerance through extended survival measures and across multiple host settings. If our laboratory findings reflect traits of *E. postvittana* overwintering populations, our work has relevance to refined forecasts of population distributions and dynamics in cold environments. As ectotherms, insects have life histories that are intimately linked to their surrounding temperature environments. Thus, temperature tolerances, especially to cold, constitute a primary variable in risk assessments and models of insect distributions. However, these tools currently treat invading species as “homogenous and immutable entities” (Lee, 2002), being particularly void of adaptive parameters and plant–insect interactions. Climate change is exerting a powerful influence on the distributions of insects (Deutsch et al., 2008; Huey et al., 2012) and plants (Chown et al., 2012; Kelly & Goulden, 2008) and will continue to. Accurately forecasting the impacts of a changing climate on pest species demands a clear understanding of what drives their current temperature tolerances and especially their adaptive capacities. For phytophagous insects, it is clear that host plants could substantially mediate both.

## Conflict of Interests

The authors declare no conflict of interests.

## References

[ece32564-bib-0001] Addo‐Bediako, A. , Chown, S. L. , & Gaston, K. J. (2000). Thermal tolerance, climatic variability and latitude. Proceedings of the Royal Society B‐Biological Sciences, 267, 739–745. 10.1098/rspb.2000.1065 PMC1690610 10819141

[ece32564-bib-0002] Andersen, L. H. , Kristensen, T. N. , Loeschcke, V. , Toft, S. , & Mayntz, D. (2010). Protein and carbohydrate composition of larval food affects tolerance to thermal stress and desiccation in adult *Drosophila melanogaster* . Journal of Insect Physiology, 56, 336–340. 19931279 10.1016/j.jinsphys.2009.11.006

[ece32564-bib-0003] Awmack, C. S. , & Leather, S. R. (2002). Host plant quality and fecundity in herbivorous insects. Annual Review of Entomology, 47, 817–844. 10.1146/annurev.ento.47.091201.145300 11729092

[ece32564-bib-0004] Bale, J. S. (1993). Classes of insect cold hardiness. Functional Ecology, 7, 751–753.

[ece32564-bib-0005] Bale, J. S. (1996). Insect cold hardiness: A matter of life and death. European Journal of Entomology, 93, 369–382.

[ece32564-bib-0006] Bale, J. S. (2002). Insects and low temperatures: From molecular biology to distributions and abundance. Philosophical Transactions of the Royal Society B: Biological Sciences, 357, 849–862. 10.1098/rstb.2002.1074 PMC1693004 12171648

[ece32564-bib-0007] Block, W. , Erzinclioglu, Y. Z. , & Worland, M. R. (1988). Survival of freezing in *Calliphora* larvae. Cryo‐Letters, 9, 86–93.

[ece32564-bib-0100] Brown, J.W. , Epstein, M.E. , Gilligan, T.M. , Passoa, S.C. , & Powell, J.A. (2010). Biology, identification, and history of the light brown apple moth, Epiphyas postvittana (Walker) (Lepidoptera: Tortricidae: Archipini) in California. American Entomologist, 56, 34–43.

[ece32564-bib-0008] Buergi, L. P. , Roltsch, W. , & Mills, N. J. (2011). Abundance, age structure, and voltinism of light brown apple moth populations in California. Environmental Entomology, 40, 1370–1377. 22217751 10.1603/EN11165

[ece32564-bib-0009] Bürgi, L. P. , & Mills, N. J. (2010). Cold tolerance of the overwintering larval instars of light brown apple moth *Epiphyas postvittana* . Journal of Insect Physiology, 56, 1645–1650. 20600083 10.1016/j.jinsphys.2010.06.009

[ece32564-bib-0010] Carrillo, M. A. , Kaliyan, N. , Cannon, C. A. , Morey, R. , & Wilcke, W. (2004). A simple method to adjust cooling rates for supercooling point determination. Cryo letters, 25, 155–160. 15216379

[ece32564-bib-0011] Chown, S. L. , Huiskes, A. H. L. , Gremmen, N. J. M. , Lee, J. E. , Terauds, A. , Crosbie, K. , ··· Bergstrom, D. M. (2012). Continent‐wide risk assessment for the establishment of nonindigenous species in Antarctica. Proceedings of the National Academy of Sciences of the United States of America, 109, 1–6. 10.1073/pnas.1119787109 PMC3323995 22393003

[ece32564-bib-0012] Chown, S. L. , & Sinclair, B. J. (2010). The macrophysiology of insect cold‐hardiness In Denlinger D. L. & Lee R. E. (Eds.), Low temperature biology of insects (pp. 191–222). New York: Cambridge University Press.

[ece32564-bib-0013] Chown, S. L. , Sørensen, J. G. , & Sinclair, B. J. (2008). Physiological variation and phenotypic plasticity: A response to “Plasticity in arthropod cryotypes” by Hawes and Bale. Journal of Experimental Biology, 211, 3353–3357. 18931308 10.1242/jeb.019349

[ece32564-bib-0014] Colinet, H. , Larvor, V. , Bical, R. , & Renault, D. (2012). Dietary sugars affect cold tolerance of *Drosophila melanogaster* . Metabolomics, 9, 608–622.

[ece32564-bib-0015] Convey, P. (2010). Life‐history adaptations to polar and alpine environments In Denlinger D. L. & Lee R. E. J. (Eds.), Low temperature biology of insects (pp. 297–321). Cambridge, UK: Cambridge University Press.

[ece32564-bib-0016] Costanzo, J. P. , & Lee, R. E. (2013). Avoidance and tolerance of freezing in ectothermic vertebrates. Journal of Experimental Biology, 216, 1961–1967. 23678097 10.1242/jeb.070268

[ece32564-bib-0017] Danthanarayana, W. (1975a). The bionomics, distribution and host range of the light brown apple moth, *Epiphyas postvittana* (Walk.) (Tortricidae). Australian Journal of Zoology, 23, 419–437.

[ece32564-bib-0018] Danthanarayana, W. (1975b). Factors determining variation in fecundity if the light brown apple moth, *Epiphyas postvittana* (Walk.) (Tortricidae). Australian Journal of Zoology, 23, 439–451.

[ece32564-bib-0019] Deutsch, C. A. , Tewksbury, J. J. , Huey, R. B. , Sheldon, K. S. , Ghalambor, C. K. , Haak, D. C. , & Martin, P. R. (2008). Impacts of climate warming on terrestrial ectotherms across latitude. Proceedings of the National Academy of Sciences of the United States of America, 105, 6668–6672. 18458348 10.1073/pnas.0709472105 PMC2373333

[ece32564-bib-0020] Duman, J. G. , Wu, D. , & Xu, L. (1991). Adaptations of insects to subzero temperatures. Quarterly Review of Biology, 66, 387–410.

[ece32564-bib-0021] Duman, J. G. , Xu, L. , Neven, L. G. , Thursman, D. , & Wu, D. W. (1991). Hemolymph proteins involved in insect subzero‐temperature tolerance: Ice nucleators and antifreeze proteins In Lee R. E. & Denlinger D. L. (Eds.), Insects at low temperature (pp. 94–127). New York, NY: Chapman and Hall.

[ece32564-bib-0022] Dumbleton, L. J. (1932). The apple leaf roller (*Tortrix postvittana* Walker). New Zealand Journal of Science and Technology, 14, 83–92.

[ece32564-bib-0023] Follett, P. A. , & Lower, R. A. (2000). Irradiation to ensure quarantine security for *Cryptophlebia* spp. (Lepidoptera: Tortricidae) in Sapindaceous fruits from Hawaii. Journal of Economic Entomology, 93, 1848–1854. 11142322 10.1603/0022-0493-93.6.1848

[ece32564-bib-0024] Gash, A. F. , & Bale, J. S. (1985). Host plant influences on supercooling ability of the black‐bean aphid. CryoLetters, 6, 297–304.

[ece32564-bib-0025] Geier, P. W. , & Briese, D. T. (1980). The light‐brown apple moth, *Epiphyas postvittana* (Walker): 4. Studies on population dynamics and injuriousness to apples in the Australian Capital Territory. Australian Journal of Ecology, 5, 63–93.

[ece32564-bib-0026] Hanson, A. A. , & Venette, R. C. (2013). Thermocouple design for measuring temperatures of small insects. CryoLetters, 34, 261–266. 23812316

[ece32564-bib-0101] Hawes, T.C. , & Bale, J.S. (2007). Plasticity in arthropod cryotypes. Journal of Experimental Biology, 210, 2585–2592. 17644673 10.1242/jeb.002618

[ece32564-bib-0027] Hawes, T. C. , & Wharton, D. A. (2010). Tolerance of freezing in caterpillars of the New Zealand Magpie moth (*Nyctemera annulata*). Physiological Entomology, 35, 296–300.

[ece32564-bib-0028] Holmstrup, M. , Bayley, M. , Pedersen, S. A. , & Zachariassen, K. E. (2010). Interactions between cold, desiccation, and environmental toxins In Denlinger D. L. & Lee R. E. (Eds.), Low temperature biology of insects (pp. 166–178). New York, NY: Cambridge University Press.

[ece32564-bib-0029] Huey, R. B. , Kearney, M. R. , Krockenberger, A. , Holtum, J. A. M. , Jess, M. , & Williams, S. E. (2012). Predicting organismal vulnerability to climate warming: Roles of behaviour, physiology and adaptation. Philosophical Transactions of the Royal Society B: Biological Sciences, 367, 1665–1679. 10.1098/rstb.2012.0005 PMC3350654 22566674

[ece32564-bib-0030] Hunter, M. D. , & McNeil, J. N. (1997). Host‐plant quality influences diapause and voltinism in a polyphagous insect herbivore. Ecology, 78, 977–986.

[ece32564-bib-0031] Irwin, J. T. , & Lee, R. E. (2002). Energy and water conservation in frozen vs. supercooled larvae of the goldenrod gall fly, *Eurosta solidaginis* (Fitch) (Diptera: Tephritidae). Journal of Experimental Zoology, 292, 345–350. 11857468 10.1002/jez.10082

[ece32564-bib-0032] Kelly, A. E. , & Goulden, M. L. (2008). Rapid shifts in plant distribution with recent climate change. Proceedings of the National Academy of Sciences of the United States of America, 105, 11823–11826. 18697941 10.1073/pnas.0802891105 PMC2575286

[ece32564-bib-0033] Kleynhans, E. , Conlong, D. E. , & Terblanche, J. S. (2014). Host plant‐related variation in thermal tolerance of *Eldana saccharina* . Entomologia Experimentalis et Applicata, 150, 113–122.

[ece32564-bib-0034] Koštál, V. (2010). Cell structural modifications in insects at low temperatures In Denlinger D. L. & Lee R. E. (Eds.), Low temperature biology of insects (pp. 116–140). Cambridge: Cambridge University Press.

[ece32564-bib-0035] Koštál, V. , Šimek, P. , Zahradníčková, H. , Cimlová, J. , & Štětina, T. (2012). Conversion of the chill susceptible fruit fly larva (*Drosophila melanogaster*) to a freeze tolerant organism. Proceedings of the National Academy of Sciences of the United States of America, 109, 3270–3274. 22331891 10.1073/pnas.1119986109 PMC3295325

[ece32564-bib-0036] Lavy, D. , & Verhoef, H. A. (1997). Cold hardiness in the collembolan *Orchesek cinctaunder* different food conditions. Comparative Biochemistry and Physiology, 118A, 699–704.

[ece32564-bib-0037] Lee, C. E. (2002). Evolutionary genetics of invasive species. Trends in Ecology & Evolution, 17, 386–391.

[ece32564-bib-0038] Lee, R. E. (2010). A primer on insect cold‐tolerance In Denlinger D. L. & Lee R. E. (Eds.), Low temperature biology of insects (pp. 3–25). New York: Cambridge University Press.

[ece32564-bib-0039] Lee, R. E. , & Costanzo, J. P. (1998). Biological ice nucleation and ice distribution in cold‐hardy ectothermic animals. Annual Review of Physiology, 60, 55–72. 10.1146/annurev.physiol.60.1.55 9558454

[ece32564-bib-0040] Li, Y. , Zhang, L. , Zhang, Q. , Chen, H. , & Denlinger, D. L. (2014). Host diapause status and host diets augmented with cryoprotectants enhance cold hardiness in the parasitoid *Nasonia vitripennis* . Journal of Insect Physiology, 70, 8–14. 25158026 10.1016/j.jinsphys.2014.08.005

[ece32564-bib-0041] Littell, R. C. , Milliken, G. A. , Stroup, W. W. , Wolfinger, R. D. , & Schabenberger, O. (2006). Heterogeneous variance models In Littell R. C., Milliken G. A., Stroup W. W., Wolfinger R. D. & Schabenberger O. (Eds.), SAS for Mixed Models, 2nd ed. Cary, NC: SAS Institute Inc.

[ece32564-bib-0042] Liu, Z. , Gong, P. , Heckel, D. G. , Wei, W. , Sun, J. , & Li, D. (2009). Effects of larval host plants on over‐wintering physiological dynamics and survival of the cotton bollworm, *Helicoverpa armigera* (Hübner) (Lepidoptera: Noctuidae). Journal of Insect Physiology, 55, 1–9. 18761347 10.1016/j.jinsphys.2008.07.017

[ece32564-bib-0043] Morey, A. C. , Venette, R. C. , & Hutchison, W. D. (2013). Could natural selection change the geographic range limits of light brown apple moth (Lepidoptera, Tortricidae) in North America? NeoBiota, 18, 151–156.

[ece32564-bib-0044] Nedvěd, O. (2000). Snow White and the seven dwarfs: A multivariate approach to classification of cold tolerance. CryoLetters, 21, 339–348. 12148026

[ece32564-bib-0045] Overgaard, J. , Sorensen, J. G. , & Loeschcke, V. (2010). Genetic variability and evolution of cold‐tolerance In Denlinger D. L. & Lee R. E. J. (Eds.), Low temperature biology of insects, 1st ed. (pp. 276–296). New York, USA: Cambridge University Press.

[ece32564-bib-0046] Rochefort, S. , Berthiaume, R. , Hébert, C. , Charest, M. , & Bauce, E. (2011). Effect of temperature and host tree on cold hardiness of hemlock looper eggs along a latitudinal gradient. Journal of Insect Physiology, 57, 751–759. 21356214 10.1016/j.jinsphys.2011.02.013

[ece32564-bib-0047] Röder, G. , Rahier, M. , & Naisbit, R. E. (2008). Counter‐intuitive developmental plasticity induced by host quality. Proceedings of the Royal Society B: Biological Sciences, 275, 879–885. 18198142 10.1098/rspb.2007.1649 PMC2599943

[ece32564-bib-0048] Rosenheim, J. , & Hoy, M. (1989). Confidence intervals for the Abbott's formula correction of bioassay data for control response. Journal of Economic Entomology, 82, 331–335.

[ece32564-bib-0049] Shreve, S. M. , Yi, S.‐X. , & Lee, R. E. (2007). Increased dietary cholesterol enhances cold tolerance in *Drosophila melanogaster* . Cryo letters, 28, 33–37. 17369960

[ece32564-bib-0050] Sinclair, B. J. (1999). Insect cold tolerance: How many kinds of frozen? European Journal of Entomology, 96, 157–164.

[ece32564-bib-0051] Sinclair, B. J. , Addo‐Bediako, A. , & Chown, S. L. (2003). Climatic variability and the evolution of insect freeze tolerance. Biological Reviews of the Cambridge Philosophical Society, 78, 181–195. 12803420 10.1017/s1464793102006024

[ece32564-bib-0052] Sinclair, B. J. , Ferguson, L. V. , Salehipour‐Shirazi, G. , & Macmillan, H. A. (2013). Cross‐tolerance and cross‐talk in the cold: Relating low temperatures to desiccation and immune stress in insects. Integrative and Comparative Biology, 53, 545–556. 23520401 10.1093/icb/ict004

[ece32564-bib-0053] Sømme, L. (1982). Supercooling and winter survival in terrestrial arthropods. Comparative Biochemistry and Physiology, 73A, 519–543.

[ece32564-bib-0054] Spilke, J. , Piepho, H. P. , & Hu, X. (2005). Analysis of unbalanced data by mixed linear models using the mixed procedure of the SAS system. Journal of Agronomy and Crop Science, 191, 47–54.

[ece32564-bib-0055] Storey, K. B. , & Storey, J. M. (1991). Biochemistry of cryoprotectants In Lee R. E. & Denlinger D. L. (Eds.), Insects at low temperature, 1st ed. (pp. 64–93). US, Boston, MA: Springer.

[ece32564-bib-0056] Strachan, L. A. , Tarnowski‐Garner, H. E. , Marshall, K. E. , & Sinclair, B. J. (2011). The evolution of cold tolerance in *Drosophila* larvae. Physiological and Biochemical Zoology, 84, 43–53. 21050129 10.1086/657147

[ece32564-bib-0057] Suckling, D. M. , & Brockerhoff, E. G. (2010). Invasion biology, ecology, and management of the light brown apple moth (Tortricidae). Annual Review of Entomology, 55, 285–306. 10.1146/annurev-ento-112408-085311 19728834

[ece32564-bib-0058] Tomkins, A. R. , Penman, D. R. , & Chapman, R. B. (1989). Effect of temperature and host plant on development of three species of leafrollers (Lepidoptera: Tortricidae). New Zealand Entomologist, 12, 48–54.

[ece32564-bib-0059] Trudeau, M. , Mauffette, Y. , Rochefort, S. , Han, E. , & Bauce, E. (2010). Impact of host tree on forest tent caterpillar performance and offspring overwintering mortality. Environmental Entomology, 39, 498–504. 20388280 10.1603/EN09139

[ece32564-bib-0061] Venette, R. C. , Davis, E. E. , Dacosta, M. , Heisler, H. , & Larson, M. (2003). Mini Risk Assessment‐ Light brown apple moth, Epiphyas postvittana (Walker) [Lepidoptera: Tortricidae]. USDA‐APHIS‐PPQ Cooperative Agricultural Pest Survey (CAPS) report. https://www.aphis.usda.gov/plant_health/plant_pest_info/lba_moth/downloads/epostvittanapra.pdf

[ece32564-bib-0062] Verdú, J. R. , Casas, J. L. , Lobo, J. M. , & Numa, C. (2010). Dung beetles eat acorns to increase their ovarian development and thermal tolerance. PLoS One, 5, e10114. 20404931 10.1371/journal.pone.0010114 PMC2852422

[ece32564-bib-0063] Vernon, P. , & Vannier, G. (2002). Evolution of freezing susceptibility and freezing tolerance in terrestrial arthropods. Comptes Rendus Biologies, 325, 1185–1190. 12520868 10.1016/s1631-0691(02)01536-6

[ece32564-bib-0064] Via, S. (1990). Ecological genetics and host adaptation in herbivorous insects: The experimental study of evolution in natural and agricultural systems. Annual Review of Entomology, 35, 421–446. 10.1146/annurev.en.35.010190.002225 2405772

[ece32564-bib-0065] Voituron, Y. , Mouquet, N. , de Mazancourt, C. , & Clobert, J. (2002). To freeze or not to freeze? An evolutionary perspective on the cold‐hardiness strategies of overwintering ectotherms. The American Naturalist, 160, 255–270. 10.1086/341021 18707491

[ece32564-bib-0066] Zachariassen, K. E. (1985). Physiology of cold tolerance in insects. Physiological Reviews, 65, 799–832. 3903795 10.1152/physrev.1985.65.4.799

