# Supplementary material for: Host‐mediated shift in the cold tolerance of an invasive insect
Source: Ecol Evol. 2016 Oct 20;6(22):8267–75. doi: 10.1002/ece3.2564 (PMC5108276; doi:10.1002/ece3.2564)
Supplement: Supplementary file 1 [file ECE3-6-8267-s001.docx]

**Supplementary Information**

**Supplementary Information Table 1:** Mean (±SEM) of metrics used to define the suitability of five hosts of *E. postvittana* larvae in experimental blocks. Data were collected from individuals that did not experience cold stress. Numbers in parentheses indicate sample size. Not all hosts were used in each block.

|  |  | Proportion Survival** | | |  |  |
| --- | --- | --- | --- | --- | --- | --- |
| Host | block | Hatch to  late-instar | Late-instar to pupation | Pupation to adult eclosion | Mean pupal mass (mg) | Mean total developmental time (d)* |
| Artificial diet | 1 | 0.98 ±0.017 (60) | 1.00 ±0.00 (28) | 0.97 ±0.035 (28) | 36.9 ±2.1 (27) | 37.2 ±1.5 (27) |
|  | 2 | 0.98 ±0.017 (60) | 1.00 ±0.00 (36) | 0.97 ±0.027 (36) | 38.1 ±1.9 (36) | 27.4 ±0.4 (35) |
|  | 3 | 0.97 ±0.021 (66) | 0.97 ±0.031 (32) | 0.90 ±0.053 (31) | 38.0 ±1.9 (31) | 36.0 ±0.4 (28) |
| *Malus domestica* | 3 | 0.69 ±0.053 (75) | 0.81 ±0.077 (26) | 0.71 ±0.099 (21) | 30.8 ±2.1 (18) | 39.1 ±0.9 (15) |
| *Pinus banksiana* | 1 | 0.40 ±0.063 (60) | 1.00 ±0.00 (8) | 0.88 ±0.17 (8) | 29.3 ±3.9 (8) | 49.4 ±2.5 (7) |
|  | 2 | 0.62 ±0.063 (60) | 0.94 ±0.057 (17) | 1.00 ±0.00 (16) | 33.9 ±3.4 (16) | 38.4 ±2.3 (16) |
|  | 3 | 0.15 ±0.031 (134) | 1.00 ±0.00 (8) | 1.00 ±0.00 (8) | 30.6 ±3.6 (8) | 57.8 ±2.4 (8) |
| *Populus deltoides* | 3 | 0.64 ±0.055 (75) | 0.79 ±0.083 (24) | 0.74 ±0.10 (19) | 31.0 ±2.2 (18) | 34.6 ±0.4 (14) |
| *Vitis vinifera* | 1 | 0.88 ±0.041 (60) | 1.00 ±0.00 (37) | 1.00 ±0.00 (37) | 24.8 ±1.2 (28) | 50.2 ±0.9 (37) |
|  | 2 | 0.93 ±0.032 (60) | 0.89 ±0.052 (36) | 0.88 ±0.059 (32) | 20.8 ±0.7 (32) | 49.7 ±1.6 (28) |
|  | 3 | 0.73 ±0.05 (78) | 0.67 ±0.091 (27) | 0.44 ±0.12 (18) | 28.6 ±2.7 (14) | 42.1 ±1.2 (8) |

*Time from egg hatch to adult eclosion; only measured for those that survived to adult eclosion

**Sample size indicates the number of individuals going into a given developmental period

**Supplementary Information Table 2:** Larval hosts of *E. postvittana* used in each experimental block

|  | Block 1 | Block 2 | Block 3 |
| --- | --- | --- | --- |
| Artificial diet | x | x | x |
| *Malus domestica* |  |  | x** |
| *Pinus banksiana* | x* | x* | x** |
| *Populus deltoides* |  |  | x** |
| *Vitis vinifera* | x* | x* | x* |

*Material from potted seedlings or cuttings grown in a greenhouse

**Material from stock planted in St. Paul, MN
